# The efficacy and safety of sugammadex for reversing postoperative residual neuromuscular blockade in pediatric patients: A systematic review

**DOI:** 10.1038/s41598-017-06159-2

**Published:** 2017-07-18

**Authors:** Guangyu Liu, Rui Wang, Yanhong Yan, Long Fan, Jixiu Xue, Tianlong Wang

**Affiliations:** 1 Department of Anesthesiology, Peking University First Hospital, Beijing, 100035 China; 2 Department of Anesthesiology, Xuan Wu Hospital, Capital Medical University, Beijing, 100053 China; 3 Department of Anesthesiology, Beijing Tong Ren Hospital, Capital Medical University, Beijing, 100730 China

## Abstract

The aim of this study is to evaluate the efficacy and safety of sugammadex for reversing neuromuscular blockade in pediatric patients. MEDLINE and other three Databases were searched. Randomized clinical trials were included if they compared sugammadex with neostigmine or placebo in pediatric patients undergoing surgery involving the use of rocuronium or vecuronium. The primary outcome was the time interval from administration of reversal agents to train-of-four ratio (TOFr, T4/T1) > 0.9. Incidences of any drug-related adverse events were secondary outcomes. Trial inclusion, data extraction, and risk of bias assessment were performed independently. Mean difference and relative risk were used as summary statistics with random effects models. Statistical heterogeneity was assessed by the I^2^ statistic. Funnel plot was used to detect publication bias. Ten studies with 580 participants were included. Although considerable heterogeneity (I^2^ = 98.5%) was detected in primary outcome, the results suggested that, compared with placebo or neostigmine, sugammadex can reverse rocuronium-induced neuromuscular blockade more rapidly with lower incidence of bradycardia. No significant differences were found in the incidences of other adverse events. Compared with neostigmine or placebo, sugammadex may reverse rocuronium-induced neuromuscular blockade in pediatric patients rapidly and safely.

## Introduction

Neuromuscular blocking agents (NMBA) are frequently used to facilitate endotracheal intubation and mechanical ventilation and to provide good quality surgical conditions. However, postoperative residual neuromuscular blockade may increase the risk of postoperative pulmonary diseases and respiratory complications, such as pulmonary atelectasis, decreased oxygen saturation and upper airway obstruction, which may result in reintubation in the ICU and prolong the patient’s length of stay^1, 2^.

To accelerate the recovery time from neuromuscular blockade and to prevent postoperative residual neuromuscular blockade^3, 4^, acetylcholinesterase inhibitors, the only reversal agents before sugammadex, are often administered. However, these antagonist are usually associated with bradycardia, bronchospasm and other undesirable muscarinic side effects^2^. Anticholinergic drugs that are used to relieve muscarinic side effects may only be effective when used in high doses, which comes with the possibility of other unacceptable side effects^2^. Additionally, there is an association between acetylcholinesterase inhibitors and residual blockade in both pediatric and adult patients^5–9^.

Sugammadex is a selective relaxant binding agent with a modified gamma cyclodextrin, and it is specifically designed to grab and encapsulate the aminosteroidal NMBAs such as rocuronium or vecuronium^10^. Sugammadex forms a complex with these NMBAs separates NMBAs from nicotinic receptors at the neuromuscular junction therefore resulting in the reversal of the neuromuscular blockade^11^. Several clinical studies have shown that sugammadex is a safe, effective agent for the rapid reversal of aminosteroidal neuromuscular blockade for any depth of muscle relaxation^12–21^. Since the pharmacokinetic and pharmacodynamic profiles of neuromuscular blockade are affected by different age, they may be not the same between pediatric and adult patients^22^.

To examine whether sugammadex can be used to reverse rocuronium or vecuronium in pediatric patients, we reviewed randomized controlled trials (RCTs) that compared the efficacy and safety of sugammadex with neostigmine or placebo in pediatric patients undergoing general anesthesia.

## Results

### Study selection

**Figure 1:**
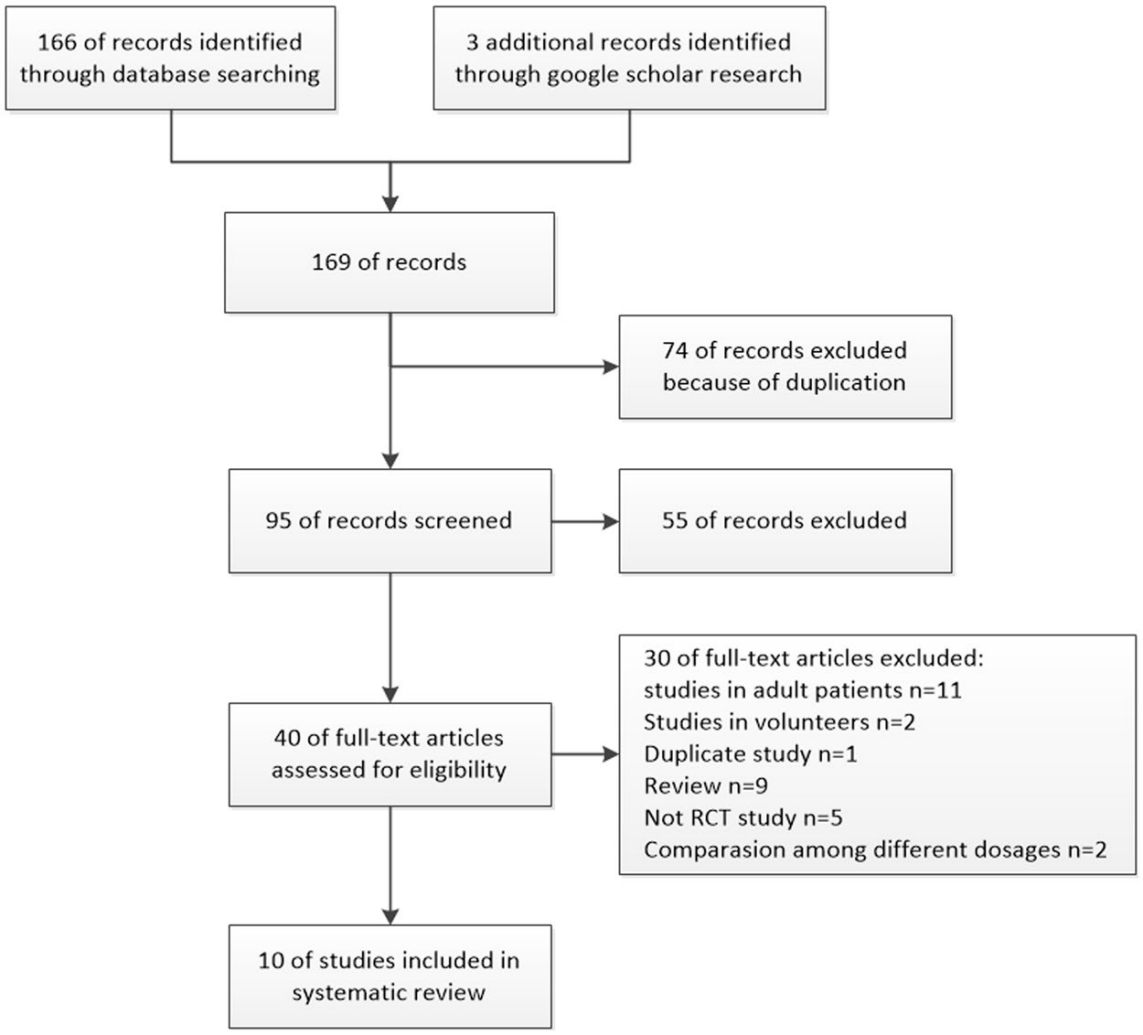
Study selection flow diagram.

We initially identified 166 studies by searching the MEDLINE, EMBASE, CENTRAL, and Web of Science databases, and three additional citations were identified through a Google Scholar search. Seventy-four citations were excluded as duplicates. Next, we scanned the abstracts of the remaining 95 citations and found that 55 did not meet our inclusion criteria. Then, we retrieved the full texts of the remaining 40 citations and excluded 30 studies. The reasons for exclusion are as follows: 13 studies were conducted using adult patients or volunteers; one study was a duplicate of another article; five studies were not RCTs; nine articles were reviews; and two studies compared different doses of sugammadex. Ten studies^23–32^ that fulfilled the criteria of our study were included. Two review authors (G.L. and R.W.) were in complete agreement regarding the inclusion of selected studies. The study selection process is shown in Fig. 1.

To obtain complete data and details to evaluate risk of bias in studies, we contacted the authors of the included studies by email. Only El sayed M replied and gave us additional details that were unreported in article.

### Study characteristics

A total of 580 eligible participants were included in the systematic review. Nine studies^24–32^ were conducted on pediatric patients over the age of 2 years, and in the study conducted by Plaud^23^, 8 infants met the eligibility criteria. Sugammadex and neostigmine were compared in nine studies^24–32^, and placebo was used as a control in the study conducted by Plaud^23^. Aside from one study^24^ that used 0.45 mg/kg of rocuronium, nine studies^23, 25–32^ used 0.6 mg/kg of rocuronium as the neuromuscular blockade agent before orotracheal intubation, and additional rocuronium was administered in seven studies^24, 27–32^. Reversal agents (sugammadex, neostigmine or placebo) were administered upon reappearance of T2 or T3 in eight studies^23, 24, 27–32^, while the effects of sugammadex on the reversal of deep rocuronium-induced neuromuscular blockade, with a post tetanic count (PTC) < 2–3, were examined in two studies^25, 26^. However, in one study^26^ reversal agents were administered at different times between the sugammadex group (PTC < 2–3) and the control group (PTC > 2–3). Inhaled anesthesia was used in six studies^24, 27–31^ and TIVA was used in one study^23^, while two studies^25, 26, 32^ did not report the maintenance of the anesthesia technique. All studies reported the time from the reversal of the neuromuscular blockade to train-of-four ratio (TOFr) > 0.9 as the primary outcome. Table 1 shows the characteristics of all of the included studies.

**Table 1:** Characteristics of included studies.

First author	year	study country	Numbers of patients	Age of patients	ASA PS	Type of surgery	Dose of NMBA	Additional NMBA	Intervention	Control	Time of reverse	Maintenance of anesthesia
Plaud B^23^	2009	Six European centers	63	28 days –17 year (2 infants, 4 children and 5 adolescents in CG/6 infants, 18 children and 23 adolescents in SG)*	1–2	Surgery in a supine position	0.6 mg/kg Roc	None	0.5 mg/kg Sug 1 mg/kg Sug 2 mg/kg Sug 4 mg/kg Sug	placebo	Reappearance of T2 three times	Opioid + Propofol
Veiga RG^24^	2011	Spain	24	2–9 years	UR	UR	0.45 mg/kg Roc	0.15 mg/kg Roc as needed	2.0 mg/kg Sug	5 mcg/kg Neo + 2.5 mcg/kg Atp**	Reappearance of T2 three times	70% N_2_O
Alvarez-Gomez JA^25^	2012	Multicenter	96	2–11 years	UR	UR	0.6 mg/kg Roc	UR	4.0 mg/kg Sug	50 mcg/kg Neo + 25 mcg/kg Atp	PTC < 2	UR
Gaona D^26^	2012	Multicenter	30	2–11 years	UR	UR	0.6 mg/kg Roc	UR	4 mg/kg Sug	50 mcg/kg Neo + 25 mcg/kg Atp	PTC < 2–3 (sug group) PTC > 2–3 (control group)	UR
Kara T^27^	2014	Turkey	80	2–12 years (5.07 ± 3.24 years in CG/6.48 ± 2.81 years in SG)	1	Lower abdominal or urogenital procedures	0.6 mg/kg Roc	0.2 mg/kg Roc as needed	2 mg/kg Sug	30 mcg/kg Neo + 10 mcg/kg Atp	Reappearance of T2	50% N_2_O + 2% Sev
Ozgun C^28^	2014	Turkey	60	2–12 years (8.0 ± 2.8 years in CG/7.3 ± 2.2 years in SG)	1–2	Ear nose and throat surgery	0.6 mg/kg Roc	0.1–0.2 mg/kg Roc as needed	2.0 mg/kg Sug	60 mcg/kg Neo + 20 mcg/kg Atp	Reappearance of T2	50% N_2_O + Sev (1.3–1.5MAC)
Ghoneim AA^29^	2015	Egypt	40	7–18years	1–3	Posterior fossa tumor excision	0.6 mg/kg Roc	0.4 mg/kg/h Roc	4 mg/kg Sug	40 mcg/kg Neo + 20 mcg/kg Atp	Reappearance of T2	0.5 μg/kg/h Fentanyl + Sev 1MAC
El sayed M^30^	2016	Egypt	70	2–10 years (5.42 ± 2.23 years in CG/5.64 ± 2.41 years in SG)	1	Tonsillectomy (outpatient)	0.6 mg/kg Roc	0.2 mg/kg Roc as needed	2.0 mg/kg Sug	50 mcg/kg Neo + 10 mcg/kg Atp	Reappearance of T2	Isoflurane
Güzelce D^31^	2016	Turkey	37	2–16 years (7.02 ± 4.46 years in CG/6.37 ± 4.08 years in SG)	1	lower urinary tract surgery and inguinal hernia	0.6 mg/kg Roc	0.15 mg/kg Roc as needed	2.0 mg/kg Sug	50 mcg/kg Neo + 20 mcg/kg Atp	Reappearance of T2	2% Sev
Mohamad Zaini RH^32^	2016	Malasia	80	2–12 years	UR	UR	0.6 mg/kg Roc	0.2 mg/kg Roc as needed	2.0 mg/kg Sug	50 mcg/kg Neo + 20 mcg/kg Atp	Reappearance of T2 or T3	UR

ASA PS: American Society of Anesthesiologists’ physical status. NMBA: neuromuscular blockade agents. UR: unreported. Roc: Rocuronium. Sug: sugammadex. Neo: neostigmine. Atp: atropine. PTC: post-titanic count. Sev: Sevoflurane. MAC: minimum alveolar concentration. CG: control group. SG: sugammadex group. *In Plaud’s study, Infant, child, and adolescent were defined as 28 days to 23 months, 2–11 years and 12–17 years, respectively. **The dose of neostigmine and atropine was suspected for that is far less than recommended.

### Risk of bias within studies

**Figure 2:**
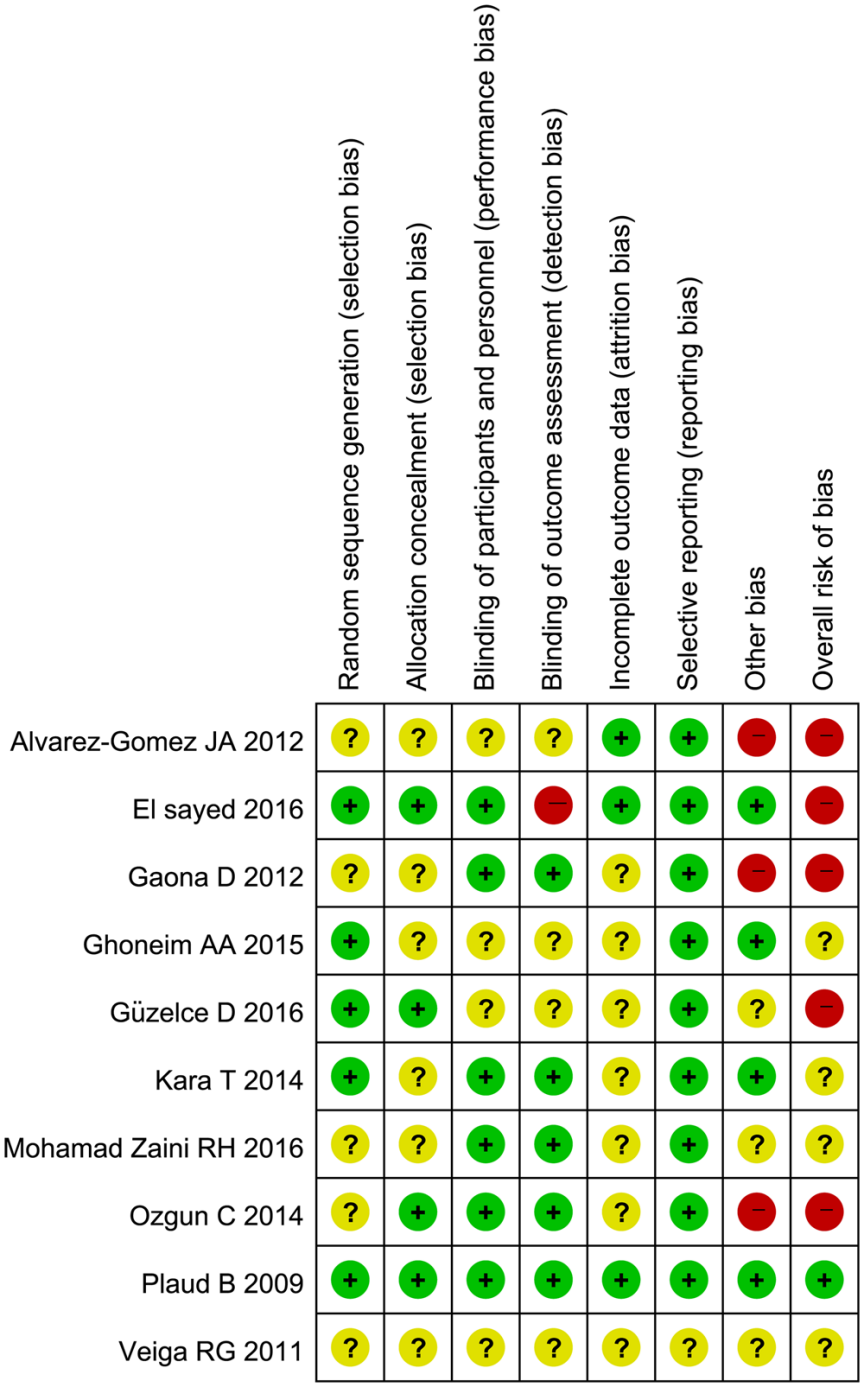
Summary of the risk of bias of the included studies.

The Cochrane Collaboration’s risk of bias tool was used to assess the validity and quality of the included Five studies^24–26, 28, 32^ were not explicit about how they generated random sequences. Six studies^24–27, 29, 32^ were unclear about their allocation concealment. Four studies^24, 25, 29, 31^ did not report whether the patients and the assessors were blinded during outcome assessments. In the study conducted by El sayed^30^, the assessors were not blinded to the allocation of groups, which may have affected the results of the study; therefore, we evaluated the study as ‘high risk’. Four studies^24, 26–28^ did not provide sufficient detail on whether there was attrition or exclusion. When we assessing risk of selective reporting in one study^24^, there was insufficient information about whether the study was considered ‘low risk’ or ‘high risk’. Since the primary outcome reports in two studies^25, 28^ were contradicted by the tables and the article text using incorrect units, we were not confident in the results and marked the study as “other risk of bias” under the high risk category. In one study^26^ reversal agents were administered at different times between the sugammadex group (PTC < 2–3) and the control group (PTC > 2–3), which we believe may have influenced the primary outcome. We believe that one trial^23^ had a low risk of bias because all of the study criteria were assessed as low risk, whereas three trials^25, 26, 28, 30, 31^ had high risks, and the risks of other studies^24, 27, 29, 32^ were unclear. Assessments of risk of bias are summarized in Fig. 2.

### Primary outcome

**Figure 3:**
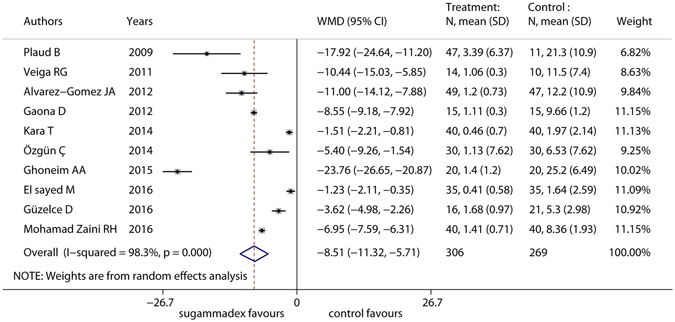
Forest plot of primary outcome of included RCTs. Sugammadex was significantly more effective than control in reducing recovery the reversal of neuromuscular blockade to TOFr > 0.9 in pediatric patients (WMD = −8.51, 95% CI: −11.32 to −5.71), but considerable heterogeneity was detected (I^2^ = 98.3%).

Primary outcome was reported in all of the studies, totaling 575 participants (protocol violations were reported for four participants, and the primary outcome of one participant was missing in one study^23^). All ten studies showed significant differences between the sugammadex group and the control group. Figure 3 shows that sugammadex was significantly more effective than the control in reducing the time from administration of reversal agents to TOFr > 0.9 in pediatric patients (WMD = −8.51, 95% CI: −11.32 to −5.71), but considerable heterogeneity was detected (I^2^ = 98.3%). Heterogeneity was not resolved with sensitivity analyses and subgroup analyses.

### Secondary outcome

Compared with neostigmine, sugammadex was able to reduce the incidence of bradycardia (RR = 0.08; 95% CI: 0.01 to 0.42), whereas no significant differences were found for the incidence of other adverse events (AEs) between the two groups, such as nausea and vomiting (RR = 0.57; 95%CI: 0.32 to 1.03), diarrhea (RR = 0.75; 95%CI: 0.03 to 17.37), and bronchospasm (RR = 0.73; 95% CI: 0.05 to 10.78). These results are shown in Table 2.

**Table 2:** The summary of reported secondary outcome (adverse events) in the included studies.

Adverse events	Number of studies	Incidence of adverse events/total number of patients		RR [95% CI]	I^2^	*P*-value	References
		Sugammadex	Control				
Nausea and vomiting	8*	27/281	25/245	0.57[0.32, 1.03]	9%	*P* = 0.355	24, 25, 27–32
Bradycardia	5*	0/190	15/149	0.08[0.01, 0.42]	*0%*	*P* = 0.823	23, 25–27, 30
Tachycardia	1	0/40	0/40	/	/	/	27
Hypotension	2	0/75	0/75	/	/	/	27, 30
QTc prolongations	2	0/91	0/52	/	/	/	23, 27
Constipation	1	2/51	0/12	1.25[0.06, 24.48]	/	/	23
Diarrhea	1	1/51	0/12	0.75[0.03, 17.37]	/	/	23
Viral gastroenteritis	1	1/51	0/12	0.75[0.03, 17.37]	/	/	23
Nasopharyngitis	1	1/51	0/12	0.75[0.03, 17.37]	/	/	23
Pharyngitis	1	1/51	0/12	0.75[0.03, 17.37]	/	/	23
Rhinitis	1	0/51	1/12	0.08[0.00, 1.93]	/	/	23
Bronchospasm	3*	1/119	2/117	0.73[0.05, 10.78]	*34*.*6%*	*P* = 0.216	25, 27, 28
Hypoglycemia	1	1/51	0/12	0.75[0.03, 17.37]	/	/	23
Pyrexia	2*	0/91	1/52	0.08[0.00, 1.93]	/	/	23, 27
Pain	1	2/51	0/12	1.25[0.06, 24.48]	/	/	23
Procedural pain	1	17/51	4/12	1.00[0.41, 2.43]	/	/	23
Diplopia	2	0/56	0/61	/	/	/	27, 31
Hyper salivation	1	0/40	0/40	/	/	/	27
Dysgeusia	1	0/40	0/40	/	/	/	27
Rash	3	0/91	0/96	/	/	/	27, 30, 31
Postoperative anemia	1	2/51	0/12	1.25[0.06, 24.48]	/	/	23

*Several studies were excluded when relative risk was calculated because the incidences of two groups were both zero.

### Publication bias

**Figure 4:**
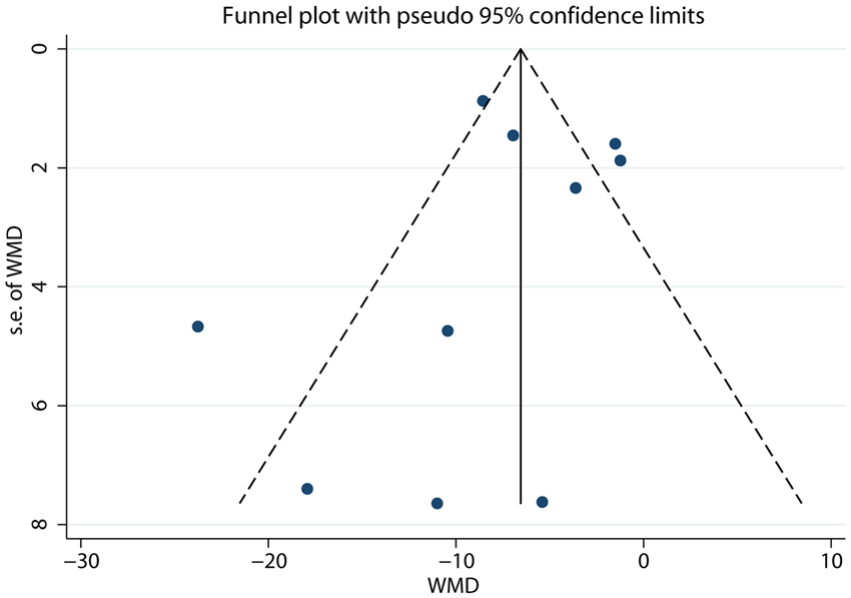
Funnel plot of primary outcome of included RCTs.

With ten studies included, a funnel plot was used to assess publication bias. Figure 4 shows that the funnel was not entirely symmetrical.

### Sensitivity analysis and subgroup analysis

Two separate sensitivity analyses were performed (Table 3) as follows: (1) excluding studies with an unclear or high risk of bias and (2) a comparison of weighted mean difference and standard mean difference.

**Table 3:** Subgroup analysis and sensitivity analysis.

Subgroups	Number of studies	Number of patients	Weighted Mean difference (or Standardized mean difference) [95% CI]	I^2^
**Risk of bias**				
Low risk	1	58	−17.92 [−24.64, −11.20]	/
High or unclear risk	9	517	−7.82 [−10.70, −4.94]	98.4%
**Intervention effect measure scale**				
Weighted mean difference	10	575	−8.51 [−11.32, −5.71]	98.3%
Standardized mean difference	10	575	−2.56 [−3.46, −1.66]	94.2%
**controls**				
neostigmine	9	517	−7.82 [−10.70, −4.94]	98.4%
placebo	1	58	−17.92 [−24.64, −11.20]	/
**Dose of sugammadex**				
4 mg/kg	4	188	−15.66 [−23.61, −7.70]	97.3%
2 mg/kg	7	373	−5.81 [−8.50, −3.12]	97.0%
1 mg/kg	1	24	−18.57[−25.15, −11.99]	/
0.5 mg/kg	1	23	−13.53[−22.71, −4.35]	/
**Time of reverse**				
T2 reappeared	8	449	−8.24 [−11.53, −4.95]	98.1%
PTC < 2–3	2	126	−9.28 [−11.47, −7.08]	56.0%

According to the protocol, subgroup analyses should be performed according to control (neostigmine or placebo), patient age (infant, child or adolescent), and neuromuscular blocker (rocuronium or vecuronium). However, because of an insufficient amount of data, subgroup analyses were performed according to (1) controls (neostigmine or placebo), (2) sugammadex dose (post-hoc analysis), and (3) time of reverse (post-hoc analysis). The results are listed in Table 3. The results showed that the I^2^ values were higher than 50% in each subgroup analysis and were higher than 90% in most situations.

### Quality of the evidence

Evidence quality of nausea and vomiting was assessed as moderate. Other outcomes were assessed as having a very low or low level of evidence quality, and the results are listed in the Supplementary Table.

## Discussion

Our study suggests that, compared with neostigmine or a placebo, sugammadex may reverse rocuronium-induced neuromuscular blockade rapidly in pediatric patients. The included studies demonstrated that sugammadex was well tolerated in the majority of pediatric patients

In this meta-analysis, the authors included all RCTs using sugammadex in pediatrics that met the inclusion criteria, regardless of the publication stage (full-text published or conference abstracts). The inclusion of conference abstracts can have advantages as well as problems. The Cochrane Handbook for Systematic Reviews^33^ recommends that grey literature (for example, conference abstracts) should be included because a systematic review of data from only published reports may present a misleading picture of an intervention’s efficacy. According to an updated Cochrane methodology review, the exclusion of grey literature may exaggerate the estimates of the intervention’s efficacy by 15% to 38%, depending on the type of grey literature^34^. Conversely, all of the included conference abstracts^24–26, 32^ adequately reported the primary and secondary outcomes, which are the time from administration of reversal agents to TOFr > 0.9 and the incidence of adverse events. However, there was inadequate detail on the methodological quality and the adverse events in abstract-only studies. To address this problem, all authors of these studies were contacted and asked for further information about their studies. Unfortunately, only one author replied to our inquiry. Due to the limited details and amount of author feedback, several items from the included studies had an unclear risk of bias.

Regarding primary outcomes, there are some issues that need to be considered before generalizing the results. First, all of the included studies used acceleromyography (AMG) to define the recovery of neuromuscular blockade as TOFr 0.9. However, several methods of quantitative monitoring have been used in clinical studies^35^. Anesthesiologists should pay attention to the differences between AMG and other methods, such as mechanomyograph (MMG) and electromyography (EMG)^35^. Second, the reported SDs in the different studies ranged widely, which cannot be explained by the design of the trials. For example, studies conducted by Alvarez-Gomez JA *et al*.^25^ and Gaona D *et al*.^26^ both examined deep blockades (PTC < 2–3) with rocuronium and compared 4 mg/kg sugammadex to neostigmine + atropine in 2 to 11 year-old patients. However, the SDs in the control groups (10.9 in the study conducted by Alvarez-Gomez JA^25^ compared to 1.2 in the study conducted by Gaona D^26^) were quite different. In these studies, unstable tracing during AMG and patient movement may partially account for the unreliable measurements.

The included studies used sugammadex to reverse in moderate (reappearance of T2 or T3) or deep (PTC < 2 or 2–3) of neuromuscular blockade, but RCTs conducted on pediatric patients with shallow (TOFr 0.25 or 0.5) or very deep (just a few minutes after high dosage of neuromuscular blockade agents) neuromuscular blockades were not identified. First, RCTs that compared the use of sugammadex with the use of neostigmine or placebo to reverse shallow neuromuscular blockades (TOFr 0.25 or 0.5) in pediatric patients were not available. Second, eight^23, 24, 27–32^ of the ten included studies compared sugammadex and neostigmine or placebo in moderate depth neuromuscular blockades (reappearance of T2 or T3). A possible cause for this high ratio is that neostigmine cannot reverse deep neuromuscular blockade and is only recommended for use in moderate depth neuromuscular blockade. Third, the included studies^25, 26^ also suggested that sugammadex safely and rapidly reversed deep (PTC < 2 or PTC < 2–3) neuromuscular paralysis following rocuronium. Finally, situations including “cannot intubate, cannot ventilate (CICV)” or failed intubation during “rapid sequence induction” are another potential application of sugammadex. Woloszczuk-Gebicka B *et al*.^36^ reported a case that CICV happened in a nine-month-old infant, and 8 mg/kg sugammadex was administered after 0.1 mg/kg vecuronium. It was reported that spontaneous breathing returned 25 s after sugammadex administration.

Research has shown that sugammadex is well tolerated in pediatric patients. The incidence of bradycardia in the sugammadex group is lower than that in the placebo or neostigmine group. Additionally, sugammadex, compared with placebo or neostigmine, did not increase the incidence of other AEs in pediatric patients. Unlike neostigmine, sugammadex does not have an anticholinesterase effect and does not require atropine or glycopyrronium; thus, it provides greater cardiovascular stability than neostigmine. On the other side, in experimental research, one study^37^ showed that clinically relevant sugammadex concentrations may cause apoptotic or neuron death in primary cultures. In an intact brain barrier this will be unlikely, however, if the brain barrier is affected by systemic infection, intracranial bleeding or head trauma, sugammadex may have negative effects on neuronal cells.

In this systematic review, a study conducted by Plaud B^23^ using eight infant patients provided limited data on sugammadex used in infants. However, several case reports^38, 39^ and a cohort study^40^ provided more data on infant patients. Two case reports^38, 39^ described the use of sugammadex in three neonates following abdominal surgery. In these reports, sugammadex rapidly reversed neuromuscular blockade without any adverse events. Alonso A *et al*.^40^ conducted a cohort study with 23 neonates (eight patients were one-day-old). Changes in vital signs were not observed after the administration of sugammadex.

The funnel plot was not perfectly symmetrical. The funnel plot suggested the possible presence of a potential publication bias, a language bias, inflated estimates by a flawed methodological design in smaller studies and/or a lack of publication of small trials with opposite results.

There are some limitations of this study, and the first limitation is its high heterogeneity. The reason for high heterogeneity in the primary outcome data may be related to the fact that volatile anesthetics, such as sevoflurane or isoflurane, can enhance the effect of neuromuscular blockade agents, thus affecting the reversal of rocuronium-induced neuromuscular paralysis. Six studies^24, 27–31^ used various concentrations of sevoflurane, isoflurane or N_2_O during operation, whereas three studies^25, 26, 32^ did not report whether volatile anesthetics were used during operation. Another limitation of this meta-analysis is that most of the included studies^24–32^ were determined to have unclear or high risk of bias, and the qualities of the primary outcome and most secondary outcomes were assessed as having low risk or very low risk.

In conclusion, compared with neostigmine or placebo, sugammadex may reverse rocuronium-induced neuromuscular blockade rapidly and safely in pediatric patients. Further studies should be conducted to help confirm the efficacy and safety of sugammadex in this special population.

## Methods

This study protocol was registered in PROSPERO 2015 and register ID is CRD42015032448. The results of this study were reported following the Preferred Reporting Items for Systematic Reviews and Meta-analyses (PRISMA) guidelines.

### Eligibility criteria

Randomized clinical trials were included if they compared sugammadex with either neostigmine or a placebo in pediatric patients who were undergoing surgery involving the use of rocuronium or vecuronium. There were no language or publication date restrictions. Studies comparing reversal with sugammadex at different doses or with placebo were included. However, studies comparing sugammadex and sugammadex combined with neostigmine were excluded. The primary outcome of our study is the time from administration of the reversal agents to TOFr > 0.9. Incidences of any drug-related AEs were analyzed as secondary outcomes.

### Search strategy

We searched the MEDLINE (PubMed), EMBASE, Cochrane Central Register of Controlled Trials (CENTRAL), and Web of Science^TM^ databases for research that was published prior to Jan 20, 2017 without any language limitations. We also checked the reference lists for reviews and additional studies. In addition, we searched Google Scholar for potentially useful studies. Furthermore, using the System for Information on Grey Literature in Europe (SIGLE) database, grey literature was searched to identify potential RCTs that we could use. The search terms that we used included sugammadex, org 25969, bridion and its Registry Number. Details of our MEDLINE (PUBMED) search strategy are provided in the Supplementary Text that can be found online.

### Trial inclusion

Eligibility assessment was performed independently by 2 reviewers (G.L. and R.W.), in an unblended, standardized manner. Disagreements between reviewers were resolved through discussion.

### Data extraction

The following data were extracted from every study: first author name; year of publication; study country; sample size; range of participant age and their American Society of Anesthesiologists (ASA) physical status; type of surgery; dose of neuromuscular blockade agents administered; dose of sugammadex and placebo or neostigmine administered; and the occasion for sugammadex and control agents being administered. We also extracted the time from administration of the reversal agents to TOFr > 0.9 and incidences of any drug-related AEs. Two reviewers (R.W. and G.L.) independently extracted all of the data mentioned above. Disagreements were resolved through discussion. To obtain complete outcome data and further details from the studies, we contacted the authors of the included studies via email.

### Risk of bias assessment

Using the guidelines provided in the Cochrane Handbook for Systematic Reviews of Interventions^33^, the two review authors independently assessed the risk of bias for the included trials. If all of the study criteria were assessed as adequate, the study was considered to have a low risk of bias. If one or more of the criteria in a trial were assessed as inadequate, then we considered the study to have a high risk of bias. The other trials were assessed as having an unclear risk of bias.

### Statistical analyses

Data on the primary outcome are continuous, and the mean difference is used as a summary statistic with random effects models. The secondary outcomes, drug-related AEs, are binary, and the relative risk is used as a summary statistic. We analyzed the data using the function Metan, Metabias in STATA software version 13 (College Station, TX) with a random-effect model.

### Heterogeneity assessment

Statistical heterogeneity was assessed with the I^2^ statistic, which estimates the percentage of total variance that derives from heterogeneity instead of variance from chance alone. If I^2^ is greater than or equal to 50% and is statistically significant, then we considered this to be evidence of substantial levels of heterogeneity, although we acknowledged that values of I^2^ ranging from 30% to 60% might also indicate moderate heterogeneity. When we found substantial levels of heterogeneity, we explored the reasons for the heterogeneity, and a sensitivity analysis was performed to analyze statistical heterogeneity. The standardized mean difference was used as a summary statistic instead of mean difference. Additionally, studies that were considered to have a low risk of bias were analyzed separately from the others.

### Publication bias test

To assess publication bias, if at least 10 studies were included, then a funnel plot was used. If not enough studies were included, then an egger test was used to detect publication bias.

### Publication bias and sensitivity analysis

If enough studies were included, subgroups analyses were performed according to control (neostigmine or placebo), patient age (infant, child or adolescent), and neuromuscular blocker (rocuronium or vecuronium).

### Quality of evidence

We used the principles of the GRADE system^41^, where appropriate to assess the quality of the primary and secondary outcomes. A summary of findings (SOF) table was constructed to present this assessment using the GRADEpro (available on gradepro.org). The GRADE approach appraises the quality of a body of evidence based on the extent to which one can be confident that an estimate of effect or association reflects the item being assessed. The quality of a body of evidence considers the study risk of bias (methodological quality), the directness of the evidence, the heterogeneity of the data, the precision of effect estimates and the risk of publication bias.

## Electronic supplementary material

Supplementary table

Supplementary information
