# Supplementary material for: The efficacy and safety of sugammadex for reversing postoperative residual neuromuscular blockade in pediatric patients: A systematic review
Source: Sci Rep. 2017 Jul 18;7:5724. doi: 10.1038/s41598-017-06159-2 (PMC5515941; doi:10.1038/s41598-017-06159-2)
Supplement: Supplementary file 1 — Supplementary table [file 41598_2017_6159_MOESM1_ESM.doc]

**Supplementary table**

The efficacy and safety of sugammadex for reversing postoperative residual neuromuscular blockade in pediatric patients: A systematic review

Guangyu Liu^1^, Rui Wang^2^, Yanhong Yan^3^, Long Fan^2^, Jixiu Xue^2^, Tianlong Wang^2^

^1^Department of Anesthesiology, Peking University First Hospital, Beijing, 100035, China

^2^Department of Anesthesiology, Xuan Wu Hospital, Capital Medical University, Beijing, 100053, China

^3^Department of Anesthesiology, Beijing Tong Ren Hospital, Capital Medical University, Beijing, 100730, China

|  | | | | | | |
| --- | --- | --- | --- | --- | --- | --- |
| **Sugammadex compared to neostigmine or placebo for postoperative residual neuromuscular blockade in pediatric patients** | | | | | | |
| **Patient or population**: postoperative residual neuromuscular blockade in pediatric patients  **Setting**:  **Intervention**: sugammadex  **Comparison**: neostigmine or placebo | | | | | | |
| Outcome  № of participants  (studies) | Relative effect  (95% CI) | **Anticipated absolute effects (95% CI)** | | | Quality | What happens |
|  |  | **Without sugammadex** | **With sugammadex** | **Difference** |  |  |
| Time from the reversal of neuromuscular blockade to train-of-four > 0.9 (Recovery time)  assessed with: TOF-Watch SX Monitoring Program  № of participants: 575  (10 RCTs) | - | The time from the reversal of neuromuscular blockade to train-of-four > 0.9 ranged across control groups from 1.64 to 25.16. | The mean time from the reversal of neuromuscular blockade to train-of-four > 0.9 in the intervention group was 8.07 min fewer (10.87 fewer to 5.26 fewer) | MD **8.51 min fewer**  (11.32 fewer to 5.71 fewer) | ⨁⨁◯◯  LOW ^a,b^ |  |
| Nausea and vomiting  № of participants: 526  (7 RCTs) | **RR 0.57**  (0.32 to 1.03) | 10.2% | **5.8%**  (3.3 to 10.5) | **4.4% fewer**  (6.9 fewer to 0.3 more) | ⨁⨁⨁◯  MODERATE ^c^ |  |
| Bradycardia  № of participants: 339  (5 RCTs) | **RR 0.08**  (0.01 to 0.42) | 10.1% | **0.8%**  (0.1 to 4.2) | **9.3% fewer**  (10 fewer to 5.8 fewer) | ⨁⨁◯◯  LOW ^d,e^ |  |
| QTc prolongations  № of participants: 143  (2 RCTs) | not estimable | 0.0% | **0.0%**  (0.0 to 0.0) | **0.0% fewer**  (0 fewer to 0 fewer) | ⨁◯◯◯  VERY LOW ^e,f,g^ |  |
| Bronchospasm  № of participants: 236  (3 RCTs) | **RR 0.73**  (0.05 to 10.78) | 1.7% | **1.2%**  (0.1 to 18.4) | **0.5% fewer**  (1.6 fewer to 16.7 more) | ⨁◯◯◯  VERY LOW ^d,e,h^ |  |
| ***The risk in the intervention group** (and its 95% confidence interval) is based on the assumed risk in the comparison group and the **relative effect** of the intervention (and its 95% CI).  **CI:** Confidence interval; **MD:** Mean difference; **RR:** Risk ratio | | | | | | |
| **GRADE Working Group grades of evidence**  **High quality:** We are very confident that the true effect lies close to that of the estimate of the effect  **Moderate quality:** We are moderately confident in the effect estimate: The true effect is likely to be close to the estimate of the effect, but there is a possibility that it is substantially different  **Low quality:** Our confidence in the effect estimate is limited: The true effect may be substantially different from the estimate of the effect  **Very low quality:** We have very little confidence in the effect estimate: The true effect is likely to be substantially different from the estimate of effect | | | | | | |

a. Risks of bias in nine studies were valued as unclear or high.

b. Included studies had considerable heterogeneity and I2 >90%.

c. Risks of bias in six studies were valued as unclear or high.

d. Risks of bias in three studies were valued as unclear or high.

e. Total population size is less than 400 (A threshold rule-of-thumb value).

f. Risk of bias of one study was tested as unclear.

g. Incidences of two groups were both zero.

h. Point estimates vary widely across studie(s).
