# Supplementary material for: The efficacy and safety of sugammadex for reversing postoperative residual neuromuscular blockade in pediatric patients: A systematic review
Source: Sci Rep. 2017 Jul 18;7:5724. doi: 10.1038/s41598-017-06159-2 (PMC5515941; doi:10.1038/s41598-017-06159-2)
Supplement: Supplementary file 2 — Supplementary information [file 41598_2017_6159_MOESM2_ESM.docx]

**Supplementary Text**

The efficacy and safety of sugammadex for reversing postoperative residual neuromuscular blockade in pediatric patients: A systematic review

Guangyu Liu^1^, Rui Wang^2^, Yanhong Yan^3^, Long Fan^2^, Jixiu Xue^2^, Tianlong Wang^2^

^1^Department of Anesthesiology, Peking University First Hospital, Beijing, 100035, China

^2^Department of Anesthesiology, Xuan Wu Hospital, Capital Medical University, Beijing, 100053, China

^3^Department of Anesthesiology, Beijing Tong Ren Hospital, Capital Medical University, Beijing, 100730, China

The search strategy in MEDLINE-PUBMED:

#1 randomized controlled trial [pt]

#2 controlled clinical trial [pt]

#3 randomized [tiab]

#4 placebo [tiab]

#5 drug therapy [sh]

#6 randomly [tiab]

#7 trial [tiab]

#8 groups [tiab]

#9 #1 OR #2 OR #3 OR #4 OR #5 OR #6 OR #7 OR #8

#10 animals [mh] NOT humans [mh]

#11 #9 NOT #10

#12 "Sugammadex" [nm] OR "Sugammadex" [all] OR "sugammadex" [all] OR "bridion" [all] OR "25969" [all] OR "361LPM2T56" [rn]

#13 pediatrics [mh] OR pediatric [tiab] OR pediatric [tw] OR juvenile[tiab] OR juvenile[tw]

#14 infant [mh] OR infant [tiab] OR infant [tw] OR neonate[tiab] OR neonate[tw] OR newborn[tiab] OR newborn [tw]

#15 child [mh] OR children [tiab] OR child [tiab] OR child[tw]

#16 adolescent [mh] OR adolescent [tiab] OR adolescent [tw]

#17 #13 OR #14 OR #15 OR #16

#18 #11 AND #12 AND #17
